# Blockade of STAT3 Signaling Contributes to Anticancer Effect of 5-Acetyloxy-6,7,8,4′-Tetra-Methoxyflavone, a Tangeretin Derivative, on Human Glioblastoma Multiforme Cells

**DOI:** 10.3390/ijms20133366

**Published:** 2019-07-09

**Authors:** Yen-Po Cheng, Shiming Li, Wan-Ling Chuang, Chia-Hsuan Li, Guan-Jun Chen, Ching-Chin Chang, Chi-Hung R. Or, Ping-Yi Lin, Chia-Che Chang

**Affiliations:** 1 Division of Neurosurgery, Department of Surgery, Yuanlin Changhua Christian Hospital, Changhua 50006, Taiwan; 2 Institute of Biomedical Sciences, National Chung Hsing University, Taichung 40227, Taiwan; 3 Hubei Key Laboratory for Processing and Application of Catalytic Materials, Huanggang Normal University, Huanggang 438000, China; 4 Department of Food Science, Rutgers University, New Brunswick, NJ 08901, USA; 5 Transplant Medicine & Surgery Research Center, Changhua Christian Hospital, Changhua 50006, Taiwan; 6 Department of Anesthesiology, Tungs’ Taichung MetroHarbor Hospital, Taichung 43503, Taiwan; 7 Department of Medical Research, China Medical University Hospital, Taichung 40447, Taiwan; 8 Department of Life Sciences, The iEGG and Animal Biotechnology Research Center, Ph.D. Program in Translational Medicine, Rong Hsing Research Center for Translational Medicine, National Chung Hsing University, Taichung 40227, Taiwan; 9 Department of Biotechnology, Asia University, Taichung 41354, Taiwan

**Keywords:** glioblastoma multiforme, STAT3, tangeretin, 5-acetyloxy-6,7,8,4′-tetramethoxyflavone, polymethoxyflavone, BCL-2, BCL-xL, apoptosis

## Abstract

Glioblastoma multiforme (GBM) is the most aggressive primary brain tumor with poor prognosis, largely due to resistance to current radiotherapy and Temozolomide chemotherapy. The constitutive activation of Signal Transducer and Activator of Transcription 3 (STAT3) is evidenced as a pivotal driver of GBM pathogenesis and therapy resistance, and hence, is a promising GBM drug target. 5-acetyloxy-6,7,8,4′-tetramethoxyflavone (5-AcTMF) is an acetylated derivative of Tangeretin which is known to exert anticancer effects on breast, colon, lung, and multiple myeloma; however, its effect on GBM remains elusive. Herein, we reported that 5-AcTMF suppressed the viability and clonogenicity along with inducing apoptosis in multiple human GBM cell lines. Mechanistic analyses further revealed that 5-AcTMF lowered the levels of Tyrosine 705-phosphorylated STAT3 (p-STAT3), a canonical marker of STAT3 activation, but also dampened p-STAT3 upregulation elicited by Interleukin-6. Notably, ectopic expression of dominant-active STAT3 impeded 5-AcTMF-induced suppression of viability and clonogenicity plus apoptosis induction in GBM cells, confirming the prerequisite of STAT3 blockage for the inhibitory action of 5-AcTMF on GBM cell survival and growth. Additionally, 5-AcTMF impaired the activation of STAT3 upstream kinase JAK2 but also downregulated antiapoptotic BCL-2 and BCL-xL in a STAT3-dependent manner. Moreover, the overexpression of either BCL-2 or BCL-xL abrogated 5-AcTMF-mediated viability reduction and apoptosis induction in GBM cells. Collectively, we, for the first time, revealed the anticancer effect of 5-AcTMF on GBM cells, which was executed via thwarting the JAK2-STAT3-BCL-2/BCL-xL signaling axis. Our findings further implicate the therapeutic potential of 5-AcTMF for GBM treatment.

## 1. Introduction

Glioblastoma multiforme (GBM), also known as malignant astrocytoma, Grade IV astrocytoma or glioblastoma, is the most prevailing malignant tumor of the central nervous system [1,2]. GBM is a brain tumor of high infiltration, rapid evolution, and high aggression, with an incidence of 3.21 per 100,000 populations from 2011 to 2015 in USA [3]. GBM treatments include surgical resection, radiotherapy and adjuvant Temozolomide chemotherapy [4]. Nevertheless, full resection of GBM tumors is often difficult, and poor response to radiotherapy or chemotherapy frequently occurs. As a result, GMB shows high rates of recurrence, and the 5-year survival rate is lower than 10% [5]. Therefore, novel therapeutics for GBM treatment are in urgent demand.

Signal transducer and activator of transcription 3 (STAT3) has recently emerged as a pivotal oncogenic driver of GBM because of its contribution to GBM genesis, malignant progression, stemness, and resistance to radiotherapy and Temozolomide chemotherapy [6,7,8,9,10,11,12]. Consistent with its role as a GBM driver, constitutive STAT3 activation, evidenced by the Tyrosine 705- or Serine 727-phosphorylation status of STAT3, represents an independent marker for poor prognosis of GBM [6,13,14]. Notably, preclinical evidence has substantiated the therapeutic potential of STAT3 inhibition, either by RNAi knockdown or pharmacological inhibitors, for GBM treatment, thereby highlighting STAT3 as a promising target for developing novel GBM therapeutics [15,16].

Polymethoxyflavones (PMFs) are health-promoting flavonoids enriched in the peels of citrus species, such as mandarin and sweet oranges [17,18,19]. Tangeretin (5,6,7,8,4′-pentamethoxyflavone) is the predominant PMF in tangerine peels and is known to exhibit diverse biological activities, including anti-oxidative, anti-inflammatory, and anticancer [20,21,22,23]. 5-acetyloxy-6,7,8,4′-tetramethoxyflavone (5-AcTMF) is an acetylation-modified derivative of Tangeretin which improves in vivo bioavailability and efficacy [19,24]. It is worth noting that 5-AcTMF has been demonstrated to exert in vitro or in vivo anticancer activity against a wide range of human cancers, including breast, colon, lung, and multiple myeloma [24,25,26,27]. However, the anticancer effect of 5-AcTMF on GBM has not been previously addressed.

Herein, we present evidence supporting the anticancer effect of 5-AcTMF on GBM cells. We start by demonstrating the inhibitory action of 5-AcTMF on the viability and clonogenicity of a panel of human GBM cell lines, along with 5-AcTMF-induced GBM cell apoptosis. Mechanistic investigation further identified that 5-AcTMF markedly impaired the STAT3-BCL-2/BCL-xL prosurvival signaling axis, which was responsible for the proapoptotic action of 5-AcTMF on GBM cells. For the first time, our findings reveal 5-AcTMF-elicited suppression of GBM cell growth and viability, as well as the inhibitory action of 5-AcTMF on STAT3 signaling, and further implicate the potential of including 5-AcTMF in the therapeutic regimens for GBM.

## 2. Results

### 2.1. 5-AcTMF Evoked in Vitro Cytotoxicity and Apoptosis in a Panel of Human GBM Cell Lines

**Figure 1:**
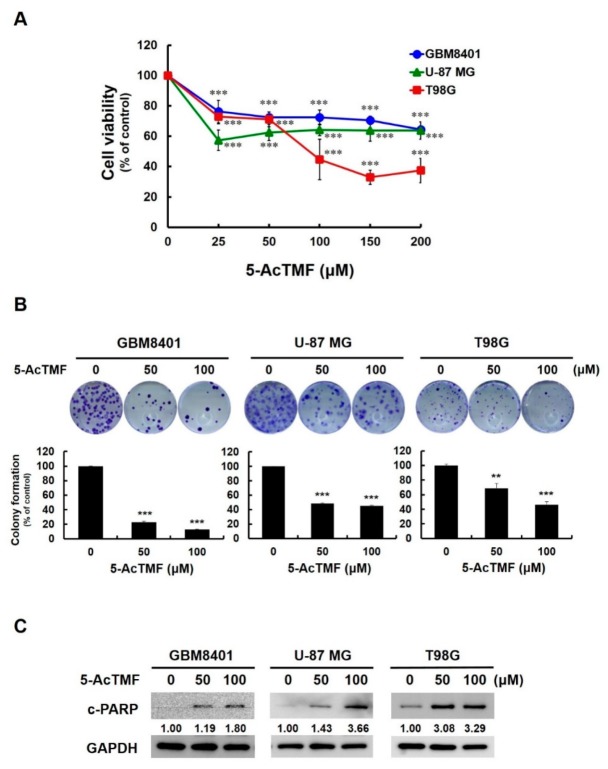
Inhibitory effect of 5-AcTMF on cell growth and survival of multiple human GBM cell lines. (**A**) Cytotoxic effect of 5-AcTMF on GBM cells. Human GBM cell lines GBM8401, U-87 MG, and T98G were treated with various concentrations (0, 25, 50, 100, and 200 μM) of 5-AcTMF for 48 h, followed by MTS assay for evaluation of cell viability. Data were represented as mean ± SD from three independent experiments. The statistic difference between 5-AcTMF-treated and drug-untreated cells were indicated (***: *p* < 0.001). (**B**) Colony formation capacity of 5-AcTMF-treated GBM cells. Colonies of GBM cell lines were revealed by crystal violet staining and then scored 10 days after 5-AcTMF treatment. Data were represented as mean ± SD from three independent experiments (**: *p* < 0.01; ***: *p* < 0.001). (**C**) Proapoptotic effect of 5-AcTMF on GBM cells. All GBM cell lines were subject to immunoblot analysis for the levels of cleaved PARP (c-PARP) following 24 h-treatment with 5-AcTMF. The levels of GAPDH were used as a loading control. The numbers beneath c-PARP blots indicate the relative density of each c-PARP immunoblot signal compared to that of 5-AcTMF-untreated control.

To evaluate whether 5-AcTMF exhibits anti-GBM activity, three human GBM cell lines, i.e., GBM8401, U-87 MG, and T98G, were exposed to graded concentrations of 5-AcTMF (0, 50, 100, 150, and 200 μM) for 48 h, and their viability was determined by MTS assay thereafter. We observed that 5-AcAMF treatment clearly led to a decline in the viability of all three cell lines compared to drug-untreated controls (*p* < 0.001), where T98G cells seemed to be most sensitive (Figure 1A). To further confirm the cytotoxic action of 5-AcTMF against GBM cells, all three GBM cell lines were subjected to analysis for their colony formation capacity after 5-AcTMF treatment (0, 50, and 100 μM). It is noteworthy that the clonogenicty of GBM8401 and U-87 MG cells was already reduced by more than 50% when treated with 50 μM of 5-AcTMF (*p* < 0.001), and the clonogenicity of T98G was lowered to 46.5 ± 3.51% of drug-free control by 5-AcTMF at 100 μM (*p* < 0.001) (Figure 1B). Thus, these results verified the inhibitory effect of 5-AcTMF on the long-term survival and proliferation of GBM cells. Lastly, to reveal the nature of 5-AcTMF-elicited cytotoxicity, the levels of PARP cleavage, a canonical hallmark of apoptosis, in 5-AcTMF-treated GBM cells were determined by immunoblotting. It was noted that a marked elevation of PARP cleavage was induced in all 5-AcTMF-treated GBM cell lines, indicating that the induction of apoptosis accounted for 5-AcTMF-elicited GBM cytotoxicity (Figure 1C; Supplementary Figure S1A). Collectively, our results demonstrated for the first time the inhibitory action of 5-AcTMF against GBM cell survival and proliferation, as well as the proapoptotic effect of 5-AcTMF on GBM cells.

### 2.2. 5-AcTMF Suppressed both Constitutive and IL-6-Induced STAT3 Activation in Human GBM cells

**Figure 2:**
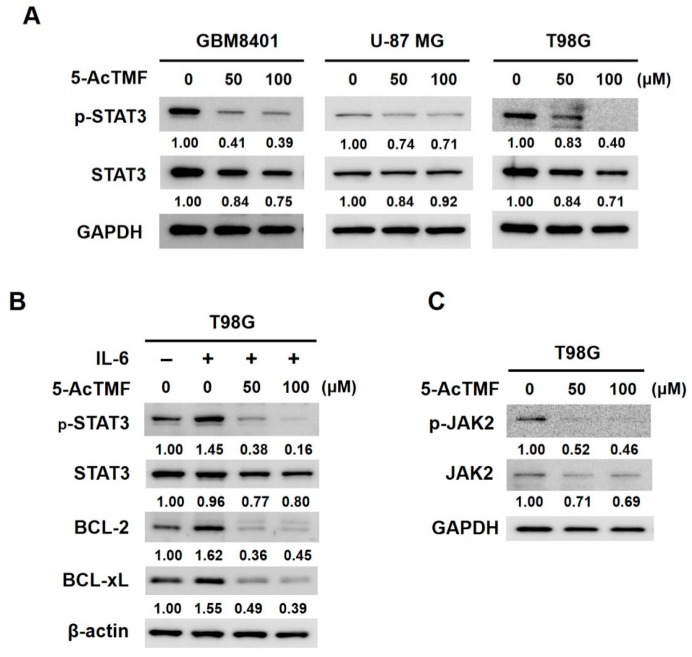
5-AcTMF suppresses constitutive and inducible activation of STAT3 in GBM cells. (**A**) 5-AcTMF lowers the levels of Tyrosine 705-phosphorylated STAT3 (p-STAT3) levels in GBM cell lines. GBM8401, U-87 MG, and T98G cells were treated with indicated concentrations of 5-AcTMF for 24 h, followed by immunoblotting for the levels of p-STAT3 and total STAT3. (**B**) Blockage of IL-6-induced STAT3 activation in GBM cells by 5-AcTMF. T98G cells were treated with 5-AcTMF for 24 h without or with stimulation by IL-6 (200 ng/mL) for 30 min. The levels of p-STAT3, total STAT3, BCL-2, and BCL-xL were examined by immunoblotting thereafter. (**C**) Inhibition of JAK2 activation by 5-AcTMF. T98G cells after 24 h-treatment with 5-AcTMF were subjected to immunoblotting for the levels of Tyrosine 1007/1008-phophorylated JAK2 (p-JAK2) and total JAK2. Either GAPDH or β-actin were used as the control for equal loading.

We next aimed to elucidate the mechanism of action underlying the anti-GBM effect of 5-AcTMF. It is noteworthy that increasing evidence has underscored persistent activation of STAT3 as a key driver for GBM tumorigenesis, progression, stemness, and resistance to radio- and chemo-therapy [17,18,19,20,21,22,23]. Along this line, we examined the effect of 5-AcTMF on the status of STAT3 activation in GBM cells. We found that in all GBM cell lines tested, 5-AcTMF markedly reduced the levels of Tyrosine 705-phosphorylated STAT3 (p-STAT3), a surrogate marker of STAT3 activation (Figure 2A; Supplementary Figures S1B and S2). This result illustrated that 5-AcTMF was able to impair constitutive STAT3 activation in GBM cells. To further support the negative effect of 5-AcTMF on STAT3, we probed whether 5-AcTMF also impedes STAT3 activation induced by interleukin 6 (IL-6). Indeed, IL-6 stimulation promoted STAT3 activation, as evidenced by IL-6-induced upregulation of p-STAT3, as well as STAT3 downstream targets BCL-2 and BCL-xL; remarkably, 5-AcTMF almost nullified IL-6-induced upregulation of p-STAT3, BCL-2 and BCL-xL (Figure 2B). These lines of evidence, taken together, substantiate the inhibitory effect of 5-AcTMF on both constitutive and inducible STAT3 activation in GBM cells.

Intriguingly, the notion that 5-AcTMF intervenes IL-6 to activate STAT3 suggested the possibility that 5-AcTMF functions at the step upstream of STAT3 phosphorylation. In light of this, we examined whether 5-AcTMF suppresses STAT3 activation via inhibiting the activities of JAK2, a canonical upstream kinase for STAT3 phosphorylation. As shown in Figure 2C, 5-AcTMF markedly lowered the levels of Tyrosine 1007/1008-phosphorylated JAK2 (p-JAK2), suggesting that inhibition of JAK2 by 5-AcTMF is likely responsible for the 5-AcTMF-mediated suppression of STAT3 activation.

### 2.3. Suppression of STAT3 Activation is Essential for 5-AcTMF to Inhibit GBM Cell Survival and Growth

**Figure 3:**
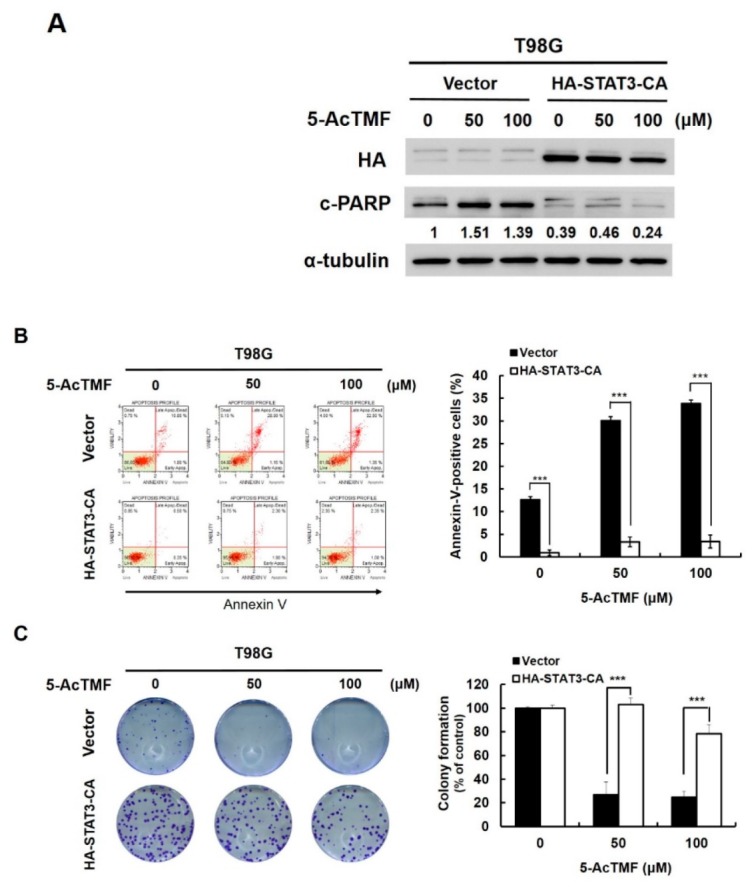
Essential role of STAT3 suppression in the anti-GBM effect of 5-AcTMF. (**A**) Constitutive STAT3 activation impaired 5-AcTMF-elicited apoptosis. T98G clones stably carrying an empty vector or the vector expressing an N-terminal HA-tagged dominant-active STAT3 mutant (HA-STAT3-CA) were treated with 5-AcTMF for 24 h, followed by immunoblotting for the levels of HA (to confirm the expression of HA-STAT3-CA) and cleaved PARP (c-PARP). α-tubulin was used as a loading control. (**B**) Blockage of STAT3 activation is required for the proapoptotic action of 5-AcTMF on GBM cells. T98G vector or STAT3-CA stable clones were treated with 5-AcTMF for 24 h and then subjected to Annexin V/PI dual staining analysis using flow cytometry. Annexin V-positive cell populations were scored as apoptotic cells and shown as the percentage of total cell population. Data were represented as mean ± SD. (**C**) Constitutive STAT3 activation protects GBM cells from 5-AcTMF-induced suppression of clonogenicity. T98G vector or STAT3-CA stable clones were treated with 5-AcTMF and then subjected to analysis for colony formation capacity. Colony numbers were scored and shown as the percentage of colonies formed in 5-AcTMF-untreated controls. Data represented as mean ± SD. ***: *p* < 0.001.

Given that the inhibitory effect of 5-AcTMF on STAT3 was firmly established, we then tried to clarify the role of STAT3 suppression in 5-AcTMF-induced apoptosis in GBM cells. To address this question, T98G cells stably expressing a constitutively active STAT3 mutant with an N-terminal hemagglutinin (HA) tag (HA-STAT3-CA) were generated to neutralize the suppression of STAT3 by 5-AcTMF. Then, these STAT3-CA stable clones were subjected to analysis for the levels of apoptosis induced by 5-AcTMF. Immunoblotting revealed that a clear elevation of PARP cleavage was observed in 5-AcTMF-treated vector control clones, as expected; however, 5-AcTMF barely elicited PARP cleavage when STAT3 was constitutively activated (Figure 3A). Likewise, 5-AcTMF-evoked increase of Annexin V-positive (hence apoptotic) cell population was abrogated in the context of constitutive STAT3 activation (Figure 3B). In line with these findings, we further determined that persistent STAT3 activity is required to sustain the clonogenicty of 5-AcTMF-treated cells (Figure 3C). In particular, in the context of 50 μM of 5-AcTMF, persistent STAT3 activation lowered apoptotic populations by nearly 10-fold (*p* < 0.001) and almost completely restored clonogenicity (from 27.11 ± 10.64% to 103.04 ± 5.46%; *p* < 0.001). Overall, these results validated the hypothesis that STAT3 suppression is an essential mechanism underlying the anticancer effect of 5-AcTMF on GBM cells.

### 2.4. 5-AcTMF Blocks STAT3 Activation to Downregulate BCL-2 and BCL-xL

**Figure 4:**
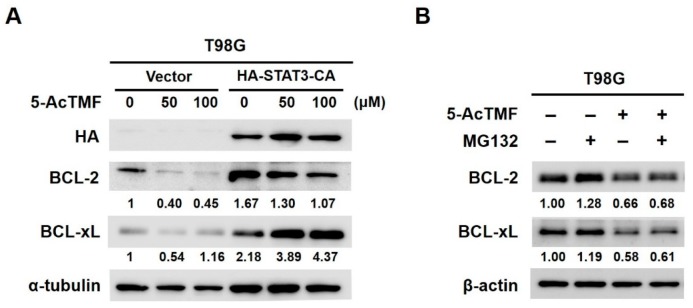
5-AcTMF downregulates antiapoptotic BCL-2 and BCL-xL through suppression of STAT3. (**A**) Constitutive STAT3 activation prevents 5-AcTMF-elicited downregulation of BCL-2 and BCL-xL. T98G vector or STAT3-CA stable clones were treated with indicated doses of 5-AcTMF for 24 h, and the levels of HA, BCL-2 and BCL-xL were determined by immunoblotting thereafter. α-tubulin was used as a loading control. (**B**) Blockage of proteasome-dependent degradation is not likely involved in 5-AcTMF-induced downregulation of BCL-2 and BCL-xL. T98G cells were treated with 100 μM of 5-AcTMF for 24 h without or with co-treatment of MG132 (20 μM) added 2 h before harvesting lysates. The levels of BCL-2 and BCL-xL in each treatment were determined using immunoblotting thereafter. β-actin served as the loading control.

Next, we aimed to define the effectors downstream of STAT3 that mediate the proapoptotic action of 5-AcTMF. We have shown in Figure 2B that 5-AcTMF curtailed the levels of IL-6-induced p-STAT3, as well as those of antiapoptotic BCL-2 and BCL-xL, both of which are known STAT3 downstream targets. Indeed, when 5-AcTMF ablated constitutive STAT3 activation, both BCL-2 and BCL-xL were clearly down-regulated as well, whereas persistent activation of STAT3 restored the levels of these two proteins (Figure 4A). On the other hand, treatment with MG132, a proteasome inhibitor, failed to rescue the levels of BCL-2 and BCL-xL reduced by 5-AcTMF (Figure 4B). Hence, our results specified that the 5-AcTMF-elicited downregulation of BCL-2 and BCL-xL is probably not achieved by evoking proteasome-mediated protein degradation, but rather, through impeding STAT3-mediated transcription.

### 2.5. BCL-2 Downregulation is Required for 5-AcTMF to Induce GBM Cell Apoptosis

**Figure 5:**
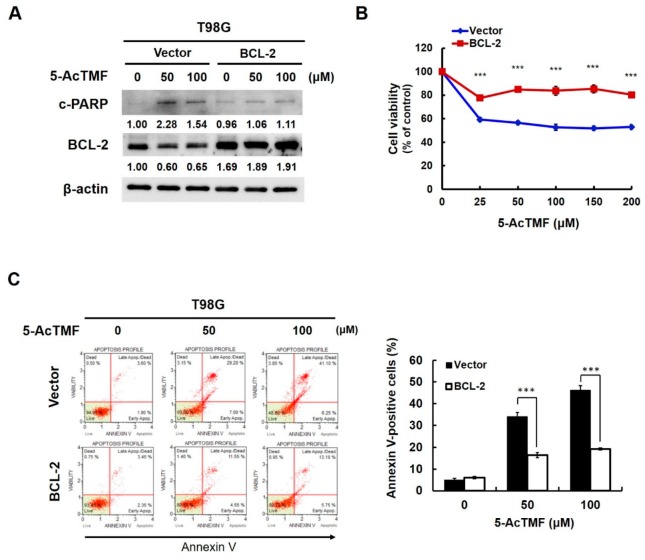
Essential role of BCL-2 downregulation in the proapoptotic effect of 5-AcTMF on GBM cells. (**A**) BCL-2 ectopic expression prevents 5-AcTMF-elicited PARP cleavage in GBM cells. T98G vector or BCL-2 stable clones were treated with indicated doses of 5-AcTMF for 24 h, and then the levels of PARP cleavage and BCL-2 were revealed by immunoblotting. β-actin was used as a loading control. (**B**) Ectopic BCL-2 expression antagonizes the cytotoxic effect of 5-AcTMF on GBM cells. Cell viability of T98G vector or BCL-2 stable clones after 24 h-treatment with 5-AcTMF (0, 50, 100 μM) was measured by MTS assay. (**C**) BCL-2 overexpression represses the proapoptotic effect of 5-AcTMF on GBM cells. T98G vector or BCL-2 stable clones treated with indicated doses of 5-AcTMF for 24 h were subjected to flow cytometry analysis for the levels of Annexin V-positive (apoptotic) cell population. ***: *p* < 0.001.

We further assessed the functional significance of BCL-2 downregulation in 5-AcTMF-elicited inhibition of GBM cell survival and growth. To this end, T98G stable clones overexpressing BCL-2 were generated and then subjected to 5-AcTMF treatment for 24 h, followed by immunoblotting for the levels of PARP cleavage. In vector clones, PARP cleavage was clearly induced by 5-AcTMF, along with BCL-2 downregulation (Figure 5A) and a marked reduction in cell viability (Figure 5B, blue line). In BCL-2-overexpressing clones, conversely, 5-AcTMF hardly provoked PARP cleavage (Figure 5A) and cytotoxicity (Figure 5B). Specifically, under 48 h-treatment with 50 μM of 5-AcTMF, the viability was enhanced by BCL-2 overexpression from 58.54 ± 1.83% to 85.01 ± 2.05% (*p* < 0.001). It is also noteworthy that BCL-2 overexpression curtailed the levels of Annexin V-positive cell population by more than 2-fold when treated with 100 μM of 5-AcTMF (*p* < 0.001) (Figure 5C). Overall, these results confirmed BLC-2 downregulation as a pivotal mechanism whereby 5-AcTMF induces GBM cell apoptosis.

### 2.6. BCL-xL Downregulation is Necessary for 5-AcTMF-Induced Apoptosis in Human GBM cells

**Figure 6:**
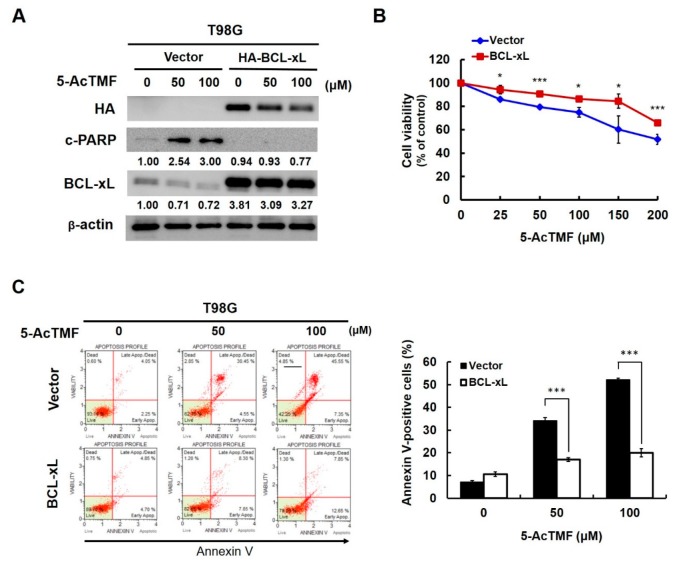
Requirement of BCL-xL downregulation for the proapoptotic effect of 5-AcTMF on GBM cells. (**A**) BCL-xL ectopic expression abolished 5-AcTMF-induced PARP cleavage in GBM cells. T98G vector or HA-BCL-xL stable clones were treated with indicated doses of 5-AcTMF for 24 h, and then subjected to immunoblotting for the levels of HA, c-PARP and BCL-xL. β-actin was used as the control for equal loading. (**B**) Ectopic BCL-xL expression alleviates 5-AcTMF-mediated cytotoxicity to GBM cells. T98G vector or HA-BCL-xL stable clones were exposed to indicated doses of 5-AcTMF for 24 h, followed by cell viability evaluation using MTS assay. (**C**) BCL-xL overexpression mitigates 5-AcTMF’s proapoptotic action on GBM cells. T98G vector or BCL-2 stable clones treated with 5-AcTMF (0, 50, 100 μM) for 24 h were then subjected to flow cytometry analysis for the levels of Annexin V-positive (apoptotic) cell population. ***: *p* < 0.001.

The role of BCL-xL downregulation in 5-AcTMF-mediated anti-GBM effect was also evaluated. To address this question, T98G cells were engineered to ectopically express an N-terminal HA-tagged BCL-xL to reverse BCL-xL downregulation. Our results indicated that neither PARP cleavage (Figure 6A) nor cytotoxicity (Figure 6B) was noticeably induced by 5-AcTMF whenever 5-AcTMF failed to downregulate BCL-xL. Likewise, ectopic BCL-xL expression dramatically abolished the increase of Annexin V-positive (apoptotic) cell population upon 5-AcTMF stimulation (Figure 6C). Notably, the apoptotic population elicited by 100 μM of 5-AcTMF was reduced by about 2.5 fold (from 52.23 ± 0.63% to 20.02 ± 1.82%; *p* < 0.001) when BCL-xL was overexpressed. Altogether, our findings established BCL-xL downregulation as a prerequisite for the proapoptotic effect of 5-AcTMF on GBM cells.

## 3. Discussion

In this report, we present various lines of evidence supporting the anticancer effect of 5-AcTMF on GBM cells, along with the underlying mechanisms of action. Specifically, we demonstrated that 5-AcTMF evidently suppressed cell viability and clonogenicity as well as promoted apoptosis of multiple human GBM cell lines (Figure 1). A mechanistic investigation established 5-AcTMF as an inhibitor of STAT3 activation, likely through blocking JAK2 activation (Figure 2), and further validated the essential role of STAT3 blockage in 5-AcTMF-elicited GBM cell apoptosis and suppression of GBM clonogenicity (Figure 3). Lastly, we proved that 5-AcTMF downregulated BCL-2 and BCL-xL in a STAT3-dependent manner (Figure 4), and that downregulation of BCL-2 or BCL-xL is required for 5-AcTMF to induce GBM cell apoptosis (Figure 5 and Figure 6). Collectively, we conclude that one of the pivotal mechanisms of action whereby 5-AcTMF exerts its anti-GBM effect is through targeted suppression of the antiapoptotic JAK2-STAT3-BCL-2/BCL-xL signaling axis. To the best of our knowledge, the findings about 5-AcTMF-elicited suppression of GBM cell growth and survival, as well as the inhibition of STAT3 signaling, have not been previously reported.

GBM is the most malignant form of glioma with poor prognosis, mostly due to limited response to current radio- and chemo- therapies as a result of intrinsic or acquired resistance [6]. Previous studies have determined that a number of signaling pathways, including the receptor Tyrosine kinase signaling axes, are integral to GBM pathogenesis, giving rise to the opportunity to develop therapeutics that target key signaling molecules for GBM treatment [13]. To this end, it is noteworthy that constitutive STAT3 activation has been validated as a fundamental GBM driver, and its role as a promising molecular target for GBM therapeutics has been evidenced in a number of preclinical trials [15,16]. In line with this notion, our discovery about 5-AcTMF as an STAT3 signaling suppressor confers a potential on 5-AcTMF as a promising GBM therapeutic. Moreover, given that STAT3 activation is responsible for GBM-associated resistance to radiotherapy and Temozolomide chemotherapy, a rational combination regimen can be designed, where 5-AcTMF is used to sensitize GBM cells to radiotherapy and Temozolomide chemotherapy via suppression of STAT3 signaling. Currently, the synergism of 5-AcTMF combined with Temozolomide for GBM therapy is under evaluation in our laboratory.

Aside from its well-known anti-inflammatory and anticancer effects, Tangeretin has also been proved to exhibit a number of beneficial bioactivities on the central nervous system, including alleviation of ischemic brain injury, improvement of cognitive memory, and protection of neurodegenerative diseases such as Alzheimer’s and Parkinson’s disease [28,29,30]. Notably, it is the ability to cross the blood-brain barrier (BBB) that allows Tangeretin to exert its neuropharmacological effects [31,32]. In line with this, it is reasonable to speculate that 5-AcTMF, as an acetylated derivative of Tangeretin, is ostensibly BBB permeable, and hence, is accessible to the location of brain tumors such as GBM, where 5-AcTMF thwarts constitutive STAT3 activation in tumor cells to induce GBM cell apoptosis. Accordingly, it would be interesting in the future to determine the tissue distribution and anti-GBM effect of 5-AcTMF in an in vivo orthotropic model of GBM.

It is perplexing that in our study, 5-AcTMF lowered GBM cell viability at a relatively high dosage. Intriguingly, Yuan et al. reported that in T98G cells, targeted inhibition of STAT3 by 200 nM of cucurbitacin I, a natural JAK2/STAT3-selective inhibitor, induced apoptosis accompanied by protective autophagy [33]. Given that 5-AcTMF inhibits STAT3 signaling and has also been shown to induce autophagy [26], an examination of whether 5-AcTMF elicits the protective autophagy that, in turn, blunts its own cytotoxicity against GBM cells is currently underway.

Data presented here regarding the anti-GBM effect of 5-AcTMF primarily focused on proapoptotic action of 5-AcTMF on GBM cells due to impairing the antiapoptotic STAT3-BCL-2/BCL-xL signaling axis. It is well-established that, besides promoting cell survival, STAT3 signaling drives additional oncogenic processes, including cell proliferation, migration, invasion, angiogenesis, and stemness [6,11,12,34]. Therefore, it is plausible to reason that the 5-AcTMF-elicited anti-GBM effect should not restrict the proapoptotic activity, but rather, include other mechanisms of action that are linked to STAT3 signaling. More experiments should be performed to clarify this issue in the future.

**Figure 7:**
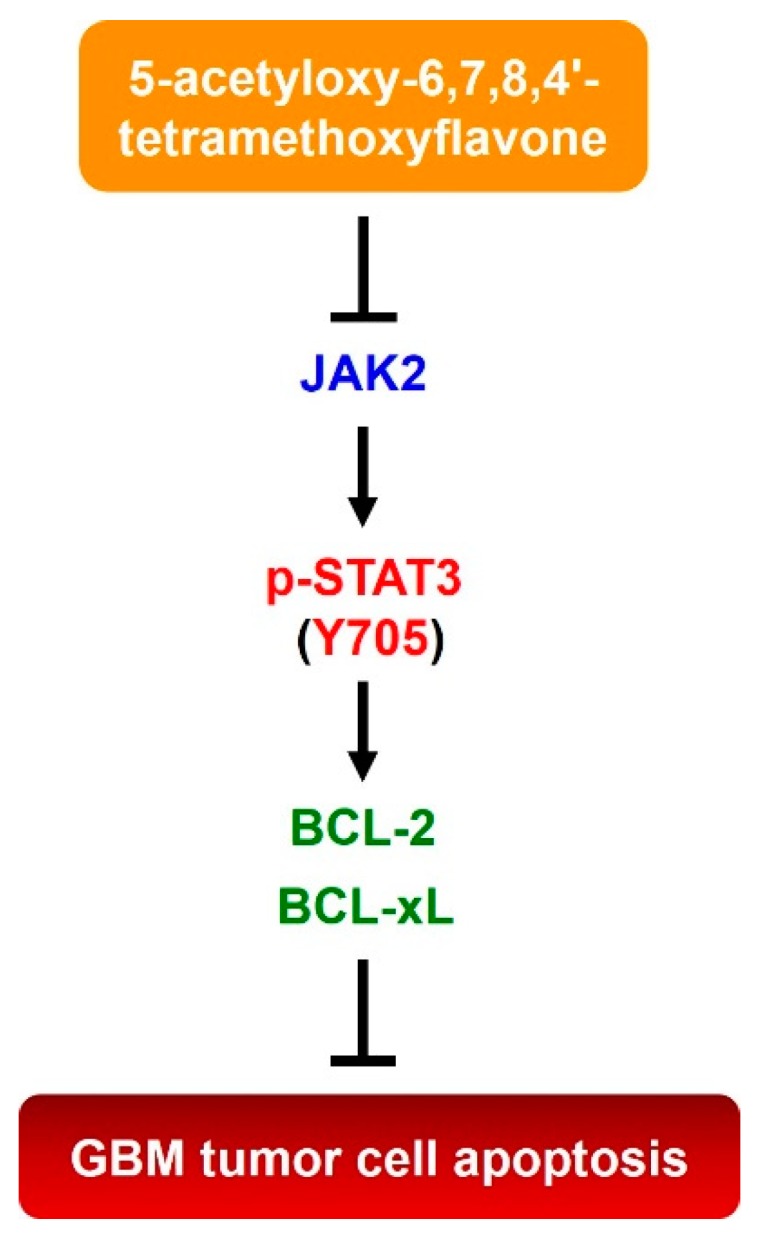
Schematic model of the mechanism of action underlying 5-AcTMF-elicited anticancer effect on GBM cells established in this study. Briefly, 5-AcTMF thwarts GBM cell survival and clonogenicity via repressing STAT3 activation, likely through inhibition of STAT3 upstream kinase JAK2, to downregulate STAT3 transcriptional targets BCL-2 and BCL-xL, thereby leading to the induction of GBM cell apoptosis.

In conclusion, we report here the newly identified anticancer effect of 5-AcTMF on GBM cells, which is mainly attributed to 5-AcTMF-mediated suppression of the JAK2-STAT3-BCL-2/BCL-xL antiapoptotic signaling axis (Figure 7). Moreover, our findings implicate the potential of applying 5-AcTMF to GBM treatment, either as a monotherapy drug or as a radio-/chemotherapies-sensitizing agent due to its inhibitory effect on STAT3 signaling.

## 4. Materials and Methods

### 4.1. Chemicals

5-AcTMF was prepared as reported previously [13,15], dissolved at a concentration of 20 mM in dimethyl sulphoxide (DMSO) (Sigma-Aldrich; St. Louis, MO, USA) as a stock solution, and stored at 4 °C until use. MG132 was obtained from AddoQ BioScience (Irvine, CA, USA). Human recombinant IL-6 was purchased from Bachem (Bubendorf, CHE). Polybrene was obtained from Sigma-Aldrich (St. Louis, MO, USA).

### 4.2. Cell Culture

T98G (ATCC^®^ CRL-1690™) and U-87 MG (ATCC^®^ HTB-14™) cell lines were purchased form the American Type Culture Collection (ATCC) (Manassas, VA, USA) and were maintained in 1× MEM medium (Gibco Life Technologies; Carlsbad, CA, USA). GBM8401 cell line was purchased from the Bioresource Collection and Research Center (Hsinchu, Taiwan) and maintained in 1× RPMI 1614 medium (Gibco Life Technologies). Both MEM and RPMI 1614 media were supplemented with 10% fetal bovine serum, 1% penicillin-streptomycin, and 1% sodium pyruvate (Gibco Life Technologies). All cells were grown at 37 °C in a humidified environment with 5% CO_2_.

### 4.3. Cell Viability Assay

Cell viability was measured using CellTiter 96^®^ AQueous One Solution Cell Proliferation Assay (MTS) according to manufacturer’s instruction (Promega; Madison, WI, USA). Briefly, cells were seeded in 96 well-plates (8 × 10^3^ cells/well), and then treated with 5-AcTMF (0, 25, 50, 100, 150, 200 μM) for 48 h. After drug treatment, 100 μL of MTS reagent was added to each reaction for 2 h-incubation, followed by measuring absorbance at 490 nm using Sunrise™ absorbance reader (Tecan, AUT). Results are expressed as a percentage of the drug-untreated controls.

### 4.4. Colony Formation Assay

Colony formation (Clonogenicity) assay was performed to assess the effect of 5-AcTMF on the long-term proliferation and survival of GBM cells. In brief, cells (4 × 10^5^) were treated with 5-AcTMF (0, 50, 100 μM) for 24 h. Then, 2 × 10^2^ of 5-AcTMF-treated cells were seeded onto 6-well plates and allowed to grow in 5-AcTMF-free culture media for 10 days for forming colonies. After that, colonies were washed with 1× PBS (Sigma-Aldrich), fixed in 100% of methanol (Sigma-Aldrich) for 15 min, and then washed with 1× PBS. Next, colonies were revealed by staining with 1% crystal violet solution (Sigma-Aldrich) for 30 min, rinsed with ddH_2_O, and then dried at room temperature (RT). The number of colonies in each well was scored using microscopy.

### 4.5. Annexin V-FITC Assay

5-AcTMF-induced apoptosis was assessed using Muse^®^ Annexin V & Dead Cell Assay Kit according to manufacturer’s instruction (Millipore; Burlington, MA, USA). Briefly, cells were seeded onto 6 well-plates (4 × 10^5^ cells/well) and then treated with 5-AcTMF (0, 50, 100 μM) for 24 h. Next, cells were collected by trypsinization, washed twice with 1× PBS and resuspended in fresh media. Lastly, cells were incubated in the dark with 100 μL of Annexin V & Dead Cell reagent for 20 min at RT, followed by flow cytometry analysis for the levels of Annexin V-positive (apoptotic) cell population using the Muse^®^ Cell Analyzer (Millipore; Burlington, MA, USA).

### 4.6. Construction of pBabe-Based Expressing Plasmids for Generating Stable Clones of HA-STAT3-CA, BCL-2, or HA-BCL-xL

The open reading frame (ORF) of the dominant-active STAT3 mutant (STAT3 (A661C/N663C); STAT3-CA) and BCL-2 were PCR-amplified using the plasmids pMXs-STAT3-C (plasmid #13373) and 3336 pcDNA3 Bcl-2 (plasmid #8768) deposited in Addgene (Cambridge, MA, USA) as the template, respectively. BCL-xL ORF was PCR-amplified from the cDNA pool of HCT 116, a human colorectal carcinoma cell line. All PCR-amplified ORFs were first TA-cloned into the pGEM-Teasy vector (Promega; Madison, WI, USA) for sequence validation. The BCL-2 ORF was later subcloned to the pBabe.puro vector (Plasmid #1764) (Addgene; Cambridge, MA, USA) at the *EcoRI* restriction site and orientation confirmed, whereas the ORFs of STAT3-CA and BCL-xL were directionally subcloned to the pBabe-HA vector, a pBabe.puro vector engineered to encode an in-frame N-terminal hemagglutinin (HA) epitope. The accession-numbers of STAT3, BCL-2 and BCL-xL mRNA are NM_139276, NM_000633, and NM_138578, respectively.

### 4.7. Retrovirus Production and Infection

293T cells, grown to approximately 70–80% confluence on 10-cm dishes, were transfected by JetPEI^TM^ (Polyplus-transfection^®^ SA; Illkirch, FRA) with 2.5 μg of the pBabe.puro empty vector, pBabe-HA-STAT3-CA, pBabe-BCL-2 or pBabe-HA-BLC-xL, together with the plasmids expressing gag-pol (2.5 μg) and VSVG proteins (0.25 μg) required for the package of viral particles. The culture media of initial transfection were collected 24 h later, followed by replacement with fresh media for additional 24 h-culture. Next, viral particles released into the culture media were harvested by centrifugation at 12,000 rpm for 3 min at 4 °C to collect the supernatant. T98G cells were then incubated for 48 h with the viral particle-enriched supernatant supplemented with 8 μg/mL of polybrene to improve infection efficiency. Next, cells with stable infection were selected with puromycin (2 μg/mL) (Sigma-Aldrich; St. Louis, MO, USA) for 48 h. Ectopic expressions of HA-STAT3-CA, BCL-2, and HA-BCL-xL were then confirmed by Immunoblotting.

### 4.8. Immunoblotting

Cells (4 × 10^5^) were treated with 5-AcTMF (0, 50, 100 μM) for 24 h, or treated with 5-AcTMF for 22 h and then added with MG132 (20 μM) for an additional 2 h. After drug treatment, cells were lysed in 100 μL of ice-cold RIPA buffer for 10 min on ice, and then spun down at 10,500 rpm for 15 min at 4 °C to collect the supernatant. Protein concentrations in the supernatant were determined by Bio-rad protein assay kit (Bio-rad Laboratories; Hercules, CA, USA). Protein samples were mixed with the sample buffer, and then denatured by heating at 95 °C for 5 min. Equal amounts (20–30 μg) of protein samples were subjected to SDS-PAGE electrophoresis, followed by transfer to polyvinylidene difluoride (PVDF) membranes (Millipore; Burlington, MA, USA). Membranes were blocked with 5% non-fat dry milk in 1× TBST buffer at RT for 1 h, and next probed with primary antibodies at 4 °C for more than 12 h. After that, membranes were washed thoroughly with 1× TBST buffer, and then probed with secondary antibodies (Jackson ImmunoResearch Laboratories Inc., West Grove, PA, USA) at RT for 1 h. Lastly, protein bands on the blot were revealed by incubation with ECL reagents (Millipore; Burlington, MA, USA), and then visualized by exposing blots to the LAS3000 system (Fujifilm; Tokyo, JPN). Densitometry analysis was performed using ImageJ software (National Institute of Health, Bethesda, MD, USA). Primary antibodies against BCL-2 (#2872), BCL-xL (#2762), HA-tag (#3724), JAK 2 (#3230), STAT3 (#12640) and c-PARP (#9541) were purchased from Cell signaling Technology (Boston, MA, USA); α-tubulin (GTX102078), β-actin (GTX109639) and GAPDH (GTX627408) were obtained from Genetex (Irvine, CA, USA); p-JAK2 (ab32101) and p-STAT3 (ab76315) were obtained from Abcam (Cambridge, GBR).

### 4.9. Statistical Analysis

All data were obtained from three independent experiments and expressed as means ± SD. An unpaired, two-tailed *t* test was performed to analyze the statistical differences between two independent groups. *p* < 0.05 was considered statistically significant.

## Supplementary Materials

The following are available online at https://www.mdpi.com/1422-0067/20/13/3366/s1.
