# Supplementary material for: Blockade of STAT3 Signaling Contributes to Anticancer Effect of 5-Acetyloxy-6,7,8,4′-Tetra-Methoxyflavone, a Tangeretin Derivative, on Human Glioblastoma Multiforme Cells
Source: Int J Mol Sci. 2019 Jul 9;20(13):3366. doi: 10.3390/ijms20133366 (PMC6651290; doi:10.3390/ijms20133366)
Supplement: Supplementary file 1 [file ijms-20-03366-s001.pdf]

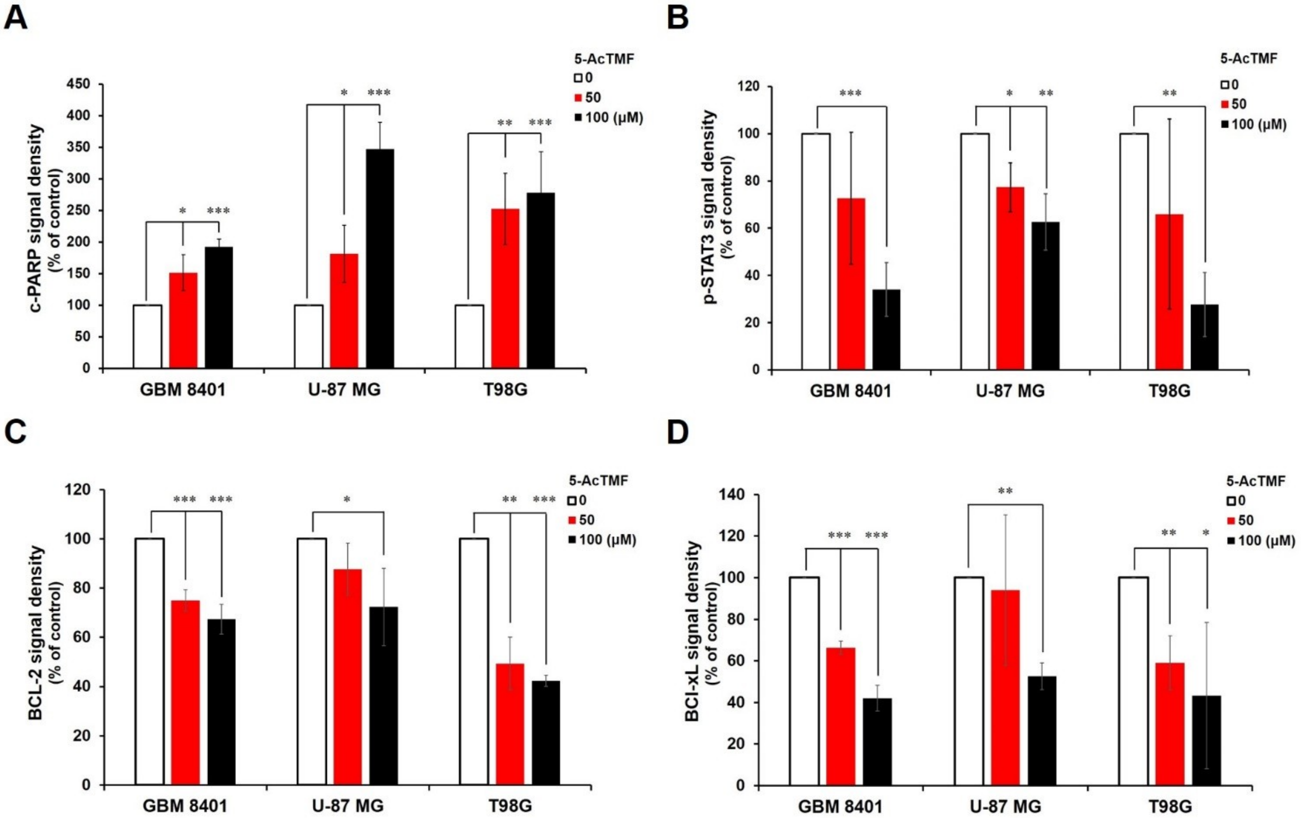

Supplementary Figure S1. Quantitation analysis for the effect of 5-AcTMF on PARP cleavage, STAT3 phosphorylation, BCL-2, and BCL-xL based upon immunoblotting. The levels of the immunoblot signal density of (A) PARP cleavage (c-PARP), (B) Tyrosine 705-phosphorylated STAT3 (p-STAT3), (C) BCL-2, and (D) BCL-xL in GBM 8401, U-87 MG and T98G cells treated with indicated dosage of 5-AcTMF (0, 50, and 100 μM) were determined using ImageJ algorithm (NCBI; Bethesda, MD, USA). Data were represented as mean ± SD from three independent immunoblots for each experimental setting. *: $p < 0.05$; **: $p < 0.01$; ***: $p < 0.001$.

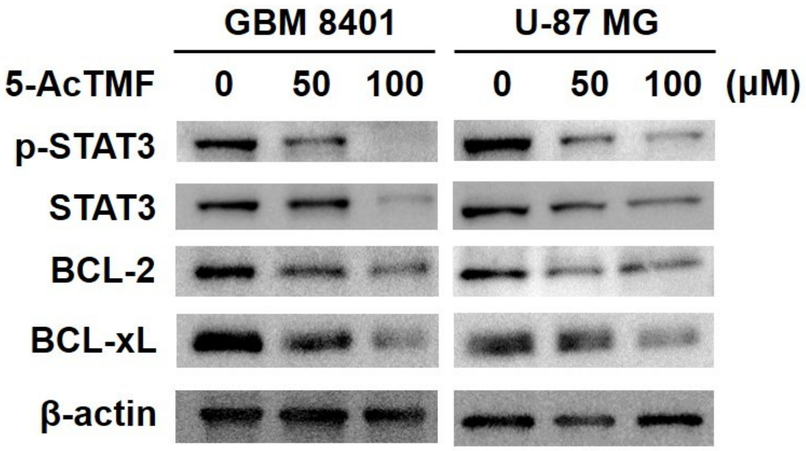

Supplementary Figure S2. In addition to T98G cells, 5-AcTMF downregulates Tyrosine 705-phosphorylated STAT3 (p-STAT3), BCL-2 and BCL-xL in GBM 8401 and U-87 MG cell lines. Human GBM cell lines GBM 8401 and U-87 MG were treated with 5-AcTMF (0, 50, and 100 μM) for 24 h, followed by immunoblotting for the levels of p-STAT3, total STAT3, BCL-2, and BCL-xL. The levels of β-actin were used as the control for equal loading. Based upon these results, it is noteworthy that 5-AcTMF-induced downregulation of p-STAT3, as well as STAT3 transcriptional targets BCL-2 and BCL-xL, appears to be universal in human GBM cell lines, suggesting that blockade of the STAT3-BCL-2/BCL-xL signaling axis represents a general mechanism of action of 5-AcTMF to elicit GBM cell apoptosis.
